# Does Inflammation Play a Major Role in the Pathogenesis of Alzheimer's Disease?

**DOI:** 10.1007/s12017-023-08741-6

**Published:** 2023-04-07

**Authors:** Benita Wiatrak, Paulina Jawień, Adam Szeląg, Izabela Jęśkowiak-Kossakowska

**Affiliations:** 1 Department of Pharmacology, Faculty of Medicine, Wroclaw Medical University, Mikulicza-Radeckiego 2, 50-345 Wrocław, Poland; 2 Department of Biostructure and Animal Physiology, Wroclaw University of Environmental and Life Sciences, Norwida 25/27, 50-375 Wroclaw, Poland

**Keywords:** Neuroinflammation, NSAIDs, Amyloid-β, Aβ* oligomers*

## Abstract

Alzheimer's disease (AD) is a neurodegenerative disease leading to dementia for which no effective medicine exists. Currently, the goal of therapy is only to slow down the inevitable progression of the disease and reduce some symptoms. AD causes the accumulation of proteins with the pathological structure of Aβ and tau and the induction of inflammation of nerves in the brain, which lead to the death of neurons. The activated microglial cells produce pro-inflammatory cytokines that induce a chronic inflammatory response and mediate synapse damage and the neuronal death. Neuroinflammation has been an often ignored aspect of ongoing AD research. There are more and more scientific papers taking into account the aspect of neuroinflammation in the pathogenesis of AD, although there are no unambiguous results regarding the impact of comorbidities or gender differences. This publication concerns a critical look at the role of inflammation in the progression of AD, based on the results of our own in vitro studies using model cell cultures and other researchers.

## Introduction

Due to an aging population, modern society struggles with an increasing incidence of neurological diseases such as Alzheimer disease (AD) (Philippens & Langermans, 2021). The total number of people with dementia is projected to reach 152 million by 2050 (Zhang et al., 2021). AD patients suffer from cognitive impairment and memory loss. Treatment is carried out with memantine (Zhang et al., 2021) and anticholinesterase drugs (galantamine, rivastigmine and donepezil), which do not reduce the brain damage caused by the ongoing neuroinflammatory process but only restore synaptic transmission (Zanon et al., 2021). Researches are conducted all the time on the pathogenesis of AD. Scientists do not yet know how the disease, which causes memory problems, and destroys the brain, arises. Efforts continue to find an effective treatment for AD. Despite the development of many theoretically promising drugs, it has not been possible to find one that would really cure the disease.

## AD Inflammatory Mechanisms and Potential Therapeutic Strategies

In 1991, three independent research teams proposed the involvement of Aβ accumulation in the pathogenesis of AD. This hypothesis holds that Aβ deposition is the earliest event in AD progression (Schwarze et al., 2021). Aβ is generated from amyloid precursor protein (APP) by abnormal proteolytic cleavage by β- and γ-secretases (Vadukul et al., 2021). Aβ plaques can aggregate around neurons leading to the dysfunction of synapses and reducing the level of the neurotransmitter acetylcholine (Uddin et al., 2020), as well as the damage and loss of cholinergic neurons (Chen et al., 2022). In the course of progressive AD disease, Aβ in the human brain can aggregate into various forms consisting of different numbers of peptides, with different shapes, sizes, and structural configurations, and with many post-translational modifications (De et al., 2019). The progressive course of AD is also associated with an increased ratio between Aβ 1–42 and Aβ 1–40 (William et al., 2021). Typically, there is a tenfold excess of Aβ 1–40 over Aβ 1–42, but Aβ 1–42 is mainly considered to be an amyloidogenic and toxic form, showing the ability to oligomerize and fibrillate. (Kageyama et al., 2021). Aβ42 oligomers cause synaptotoxicity and memory loss (Fritzsch et al., 2021). It is therefore warranted to develop agents that can effectively inhibit the formation of Aβ oligomers or block their toxicity. These properties are characterized by ALZ-801, which slows down the progression of Alzheimer's disease by reducing amyloid deposits. A Phase 3 APOLLOE4 study is underway to evaluate the effects of the experimental drug ALZ-801 on the elderly with early-onset Alzheimer's disease and two copies of APOE4, referred to as the APOE4/4 genotype (Hey et al., 2018). ApoE4 is recognized as the strongest genetic risk factor for Alzheimer's disease. It causes increased amyloid formation due to impaired cerebral blood flow (Liao et al., 2017). In addition to this, ApoE4 promotes inflammation in brain cells (Iannucci et al., 2021).

What is more, ApoE binds to the microglia receptor, acting as its ligand, triggering the receptor on myeloid cell 2 (TREM2) (Liao et al., 2017), which inhibits the expression of pro-inflammatory chemokines and cytokines. ApoE is responsible for controlling the transition of microglia from inflammation (M1) to anti-inflammatory (M2). Directly activated microglia by the neurotoxin Aβ secretes various inflammatory molecules such as IL-1, IL-6, TNF-α, IFN-γ, free radicals, and chemokines that damage neurons (Li et al., 2020). Although astrocytes in microglia remove amyloid aggregates and necrotic and apoptotic cells from brain tissue, their excessive activation may aggravate amyloidosis, leading to aggravation of the inflammatory process, damage to neurons, and decreased synaptic functionality. In addition, aggregates of Aβ and dead cells in the brain tissue stimulate proliferation and activate microglial cells, which maintains the state of stimulation of non-specific immune response, which induces the release of chronic inflammatory mediators (Azam et al., 2021). Defective clearance of beta-amyloid (Aβ) via immune cells as well as inflammatory activation of immune cells associated with Aβ is also a key factor contributing to the pathogenesis of AD. The second important cause of AD progression, apart from the formation of soluble Aβ oligomers that cause synaptic toxicity and neurodegeneration, is the occurrence of neuroinflammation causing changes in the morphology and distribution of microglia and astrocytes, and an increase in the expression of inflammatory mediators (Philippens & Langermans, 2021). Treatment with non-steroidal anti-inflammatory drugs (NASID) reduces inflammation in the nervous system significantly improving cognitive function (Fielder et al., 2020). However, NSAIDs may be beneficial in the early stages of Alzheimer's disease. Treatment of PS19 mice with JM4, which is a 19-mer cyclic peptide derived from the first loop of human erythropoietin with immunomodulatory properties, before the onset of AD reduced the neurological deficit and reduced memory impairment. The prior anti-inflammatory therapy may reduce the progression of Alzheimer's disease in the early stages of AD (Choi et al., 2021). Also, in studies in murine AD models, nilvadipine, used in the treatment of arterial hypertension, effectively reduces inflammation, tau protein hyperphosphorylation, and improves memory (Morin et al., 2020). Elevated blood pressure may increase the risk of AD due to protein extravasation into the brain tissue, which may lead to cell damage, apoptosis, and an increase in Aβ accumulation (Crous-Bou et al., 2017). An anticancer drug Regorafenib, which reduced Ab in model ad mice (5 × FAD), inhibited LPS-induced neuritis in wild-type mice. (Han et al., 2020). Similarly, Nicotinamide (NAM) or vitamin B3 can alleviate neuronal inflammation and prevent memory deficits caused by the accumulation of Aβ1-42 (Rehman et al., 2021).

Nuclear factor-κB (NF-κB) is inflammatory factor in neurodegeneration. Many papers have described the interaction of NF-κB with various molecular factors (astrocytes, β-secretase, APOE, glutamate, miRNA and tau protein) in AD research models, e.g. in mice with AD. NF-κB inhibitors may also be a promising therapeutic option for AD in the future. Current attempts at pharmacological modulation encounter difficulties related to the insufficient specificity of the NF kB inhibitors used, as well as greater susceptibility to infections. NF-κB is central to this vicious cycle of neurodegeneration and depending on the cell type and/or combination of NF-κB subunits, the activation of NF-κB can play a dual role in either neuroprotection or neurodegeneration (Sun et al., 2022).

Moreover, recent studies suggest the participation of iron and copper accumulation in generating inflammation and intensifying the death of nerve cells (Maher, 2020). Magnetic resonance imaging (MRI) results exhibited damage to the hippocampus caused by the accumulation of iron in this brain structure in patients with AD (Langkammer et al., 2014). Aluminum promotes hyperphosphorylation and aberrant aggregation of microtubule-associated (tau) protein that causes neurofibrillary tangles (NFT), causing degeneration of cortical and hippocampal neurons (Kaur et al., 2021).

**Fig. 1:**
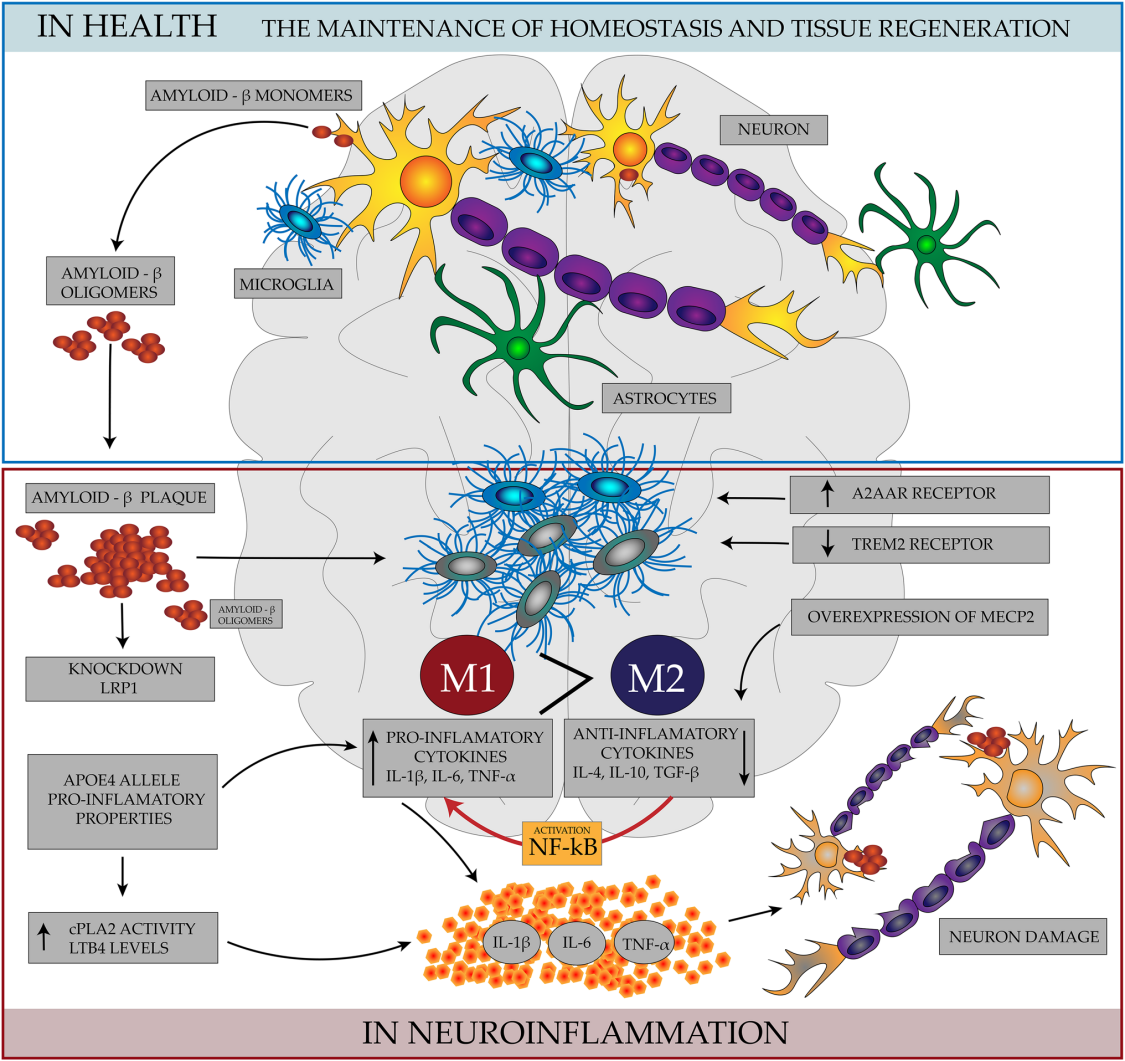
Factors causing inflammation of the nervous system

An important role in the pathomechanism of AD can also be attributed to the balance in the action of specific receptors. Microglia is regulating by the adenosine A1 and A2A (AR) receptors, considered neuroprotective and neurodegenerative receptors, respectively. A1AR is a neuroprotective receptor, and A2AAR is designated a neurodegenerative receptor. Therefore, A1AR stimulation and A2AAR inhibition may be one of the most promising treatment strategies for AD. (Marucci et al., 2021) Moreover, the peroxisome proliferator-activated PPAR-β / δ receptor, which is present in all regions of the brain, mainly neurons, has a strong anti-inflammatory effect and may stabilize the myelin sheath as well as reduce Aβ deposits. The reduction of PPAR-β / δ is responsible for the induction of inflammation of the nervous system but for the deposition of Aβ42 (Strosznajder et al., 2021). Conversely, higher levels of soluble TNFR1 and TNFR2 receptors had found in patients with AD and mild cognitive impairment (MCI). In AD, inhibition of TNF-α can reverse the effect of tau accumulation in neurites. However, long-term inhibition of TNFR1 and TNFR2 signaling enhances tau accumulation and the formation of pathological Aβ (Zhao et al., 2020) The cause of AD progression is not only dependent on soluble Aβ oligomers, which in many studies are considered to be precursors of neurodegeneration, but also on ongoing neuroinflammation and other factors mentioned. A graphical summary of all discussed factors of persistent neuroinflammation in the pathogenesis of AD is presented in Fig. 1

It cannot be unequivocally stated that the role of Aβ alone in the pathogenesis of AD is the most important. At the same time, research proves that Aβ plays a neuroprotective role at low concentrations. The studies confirmed that Aβ in low concentrations [0.001–0.1 μM] increases the growth of neurites in PC12 cultures and, at the same time, does not increase the toxicity and DNA damage in model neurobiological cultures of PC12 and THP-1. (Wiatrak & Balon, 2021; Wiatrak et al., 2022c) Another important research direction in AD is the search for neuroprotective factors, which are sought among molecules with anti-inflammatory properties. Other substances with neuroprotective and anti-inflammatory properties include two derivatives of ascorbic acid that can cross the blood–brain barrier. In addition, they also exhibit amyloid aggregation inhibition properties and antioxidant activity (Jutamas Jiaranaikulwanitch, 2021) New 4,7-dihydro-2H-pyrazolo [3-b] pyridine derivatives demonstrated strong antioxidant, anti-inflammatory, and neuroprotective properties in vitro studies (Michalska et al., 2020). The new pyrrole[3,4-d]pyridazinone derivatives improved neuronal features and may have a beneficial impact on neuronal damage caused by proinflammatory cytokines (Potyrak et al., 2021; Wakulik et al., 2020). Also, new tricyclic derivatives of 1,2-thiazine may exert neuro-regenerative effects, which has been demonstrated in a neuroinflammatory model of neuronal damage. These compounds probably cross the blood–brain barrier (BBB). The mechanism of the action of the tested compounds results from the reduction of nitrous and oxidative stress, and the inhibition of cyclooxygenase (COX) activity (Wiatrak et al., 2022b). All the tested compounds are characterized by good brain penetration and a favorable safety profile. Just like the ALZ-801 drug candidate under investigation. The ideal drug for AD must effectively cross the blood–brain barrier and reach the appropriate concentration in the brain. It should be non-toxic with anti-inflammatory activity and prevent the formation of oligomers in the case of neuroprotective drugs. In the case of the active form of AD, the ideal drug should inhibit oligomer toxicity and reduce inflammation. Unfortunately, BBB permeability is a limiting factor for many anti-inflammatory compounds. Perhaps using an appropriate drug form or targeted drug delivery technology will further improve the efficacy of test compounds. Preclinical and clinical data support the use of concentrated ultrasound (FUS) in the presence of intravenously injected microbubbles to safely and transiently increase the permeability of the blood–brain barrier (BBB). Furthermore, FUS-induced BBB permeability increases the bioavailability of drugs administered intravenously to the brain (Dubey et al., 2020). However, some anti-inflammatory compounds are also relatively large, which may limit their crossing the blood–brain barrier.

**Fig. 2:**
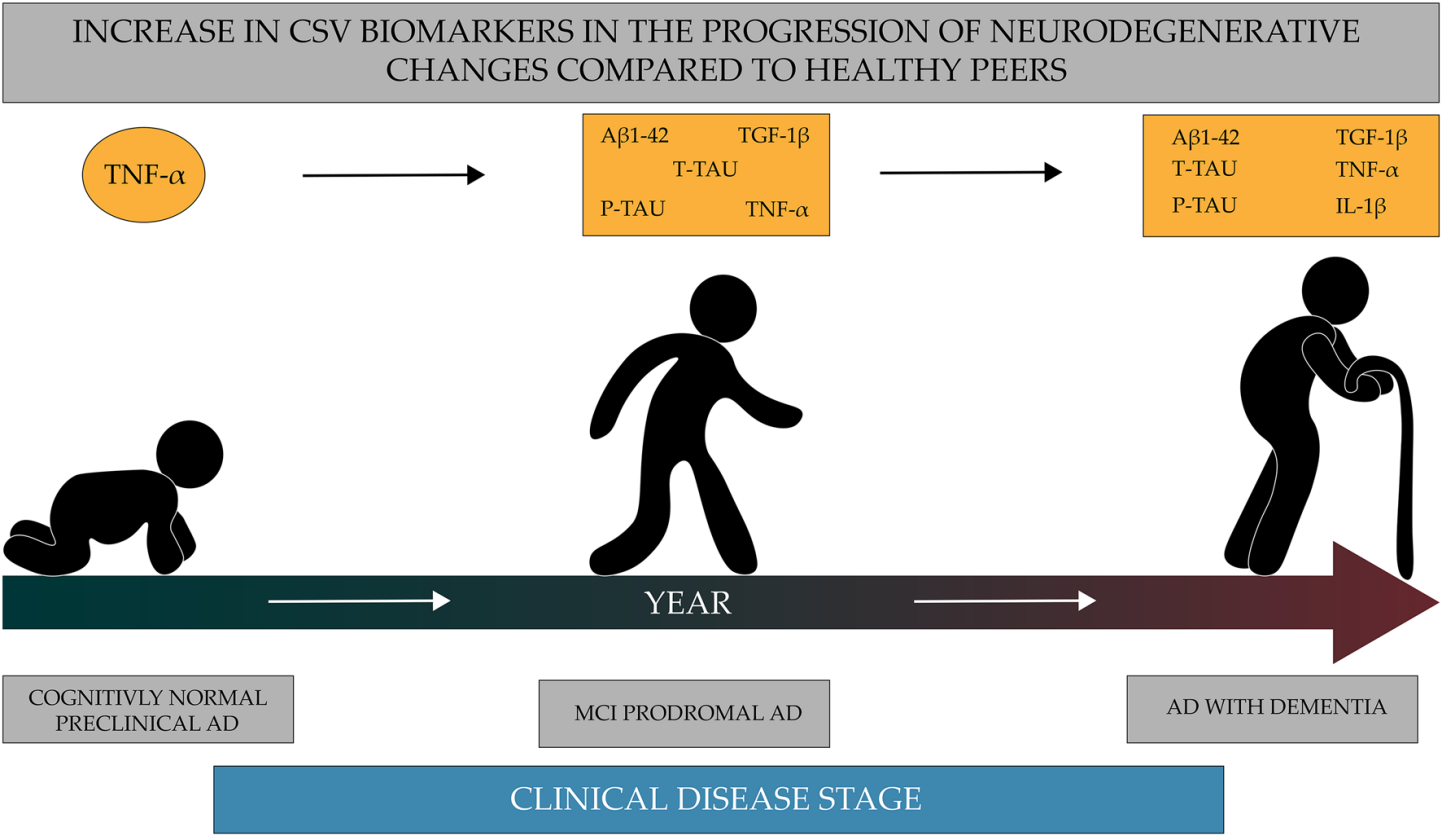
Potentially useful biomarkers in assessing the progression of neurodegenerative changes

The hope for the effective use of new anti-inflammatory compounds and already used drugs in inhibiting or delaying neurodegenerative changes in the brains of the elderly is the introduction of periodic biomarker tests: TNF-α, IL-1β, TGF-1β. The concentrations of these biomarkers increase in the serum and in the fluid of the cerebrospinal cord decades before the onset of symptoms (Fig. 2). In that case, fighting inflammation would make sense without negative effects. The anti-inflammatory compounds are also relatively large, which may limit their crossing the blood–brain barrier. It is known that with age and the progression of neurodegenerative changes, the BBB becomes unsealed. Still, if such compounds are used to be at an early stage of the disease, it may be difficult to obtaining the full pharmacological effect of a given drug.

It is worth remembering the impact of comorbidities on the progressive course of AD, where obtaining appropriate therapeutic results may be difficult. The accumulation of Aβ in the brain may increase as a result of developing diabetes (Takeuchi et al., 2019). The results of other studies indicated that insulin has a beneficial effect on cognitive functions in patients with dementia, while also having a neuroprotective effect. In contrast, metformin, an oral insulin sensitizer, increases the accumulation of Aβ therefore, its use may favor the development of AD (Picone et al., 2015). Also, a meta-analysis showed an increased risk of AD among patients with Crohn's disease and ulcerative colitis compared to the general population (Szandruk-Bender et al., 2022). In addition to this, growing evidence points to the influence of the digestive system and the disturbance of the gut microbiota to slow changes in the brain and beyond the development of AD. The intake of prebiotics, prebiotics and the Mediterranean diet inhibited the developing of dementia (Sochocka et al., 2019; Wiatrak et al., 2022a), therefore it seems that limiting AD pathomechanism only to soluble Aβ oligomers and neuroinflammation is a major simplification.

## Conclusion

Although the pathological mechanisms of amyloid beta and tau are well studied, inflammation (peripheral and central) is a novel feature of AD pathogenesis. Effective anti-inflammatory drugs are not available in advanced AD. The key to success seems to be the development of a drug that inhibits the formation or facilitates the dissolution of oligomers but does not dissolve amyloid fibrils or plaques.

## Data Availability

All data generated or analyzed during this study are included in this published article and its supplementary information files.
